# Pollen Protein Content and Developmental Success of the Solitary Bee *Osmia bicornis*: Amino Acid Thresholds for Larval Pollen Resources?

**DOI:** 10.3390/insects17030277

**Published:** 2026-03-04

**Authors:** Jordan T. Ryder, Andrew Cherrill, Helen M. Thompson, Keith F. A. Walters

**Affiliations:** 1 Agriculture and Environment Department, Harper Adams University, Newport TF10 8NB, Shropshire, UK; jordan.ryder@chester.ac.uk (J.T.R.); acherrill@harper-adams.ac.uk (A.C.); 2 Syngenta, Jealott’s Hill, Bracknell RG42 6EY, Berkshire, UK; helen.thompson@syngenta.com; 3 Department of Life Sciences, Imperial College London, Silwood Park Campus, Ascot SL5 7PY, Berks, UK

**Keywords:** pollinators, selective foraging, amino acid, nutrition, *Osmia bicornis*

## Abstract

Performance of *Osmia bicornis* larvae fed on six diets with different pollen species composition (one wild collected by foraging adults), each with known levels of nine essential amino acids (EAA; leucine, lysine, valine, arginine, isoleucine, phenylalanine, threonine, histidine, methionine), was investigated. Four of the pollen diets consisted of individual pollen species and two were mixtures of either four or five species (including one naturally foraged by adult *O. bicornis*). The diets fell into four statistically distinct groups with different EAA contents (ranked from Group I (highest EAA) to Group IV (lowest EAA; pine pollen). The highest larval survival rate was recorded with the wild-foraged diet (Group III) with no survival in Group IV. Similar survival occurred for all other diets. Where larvae survived (Group I–III), there was no effect of diet on the time to commencement of larval stages, cocoon completion or larval development time (egg hatch to pupation), or on pupal weight. The findings provide corroborative evidence of the existence of amino acid thresholds for larval success, but the need for further work is discussed in relation to their multidimensional nutritional requirements and variation of the nutritional content of pollen.

## Simple Summary

Bees provide a key ecosystem service as pollen vectors, and many flowering plants produce nutritionally rich pollen/nectar that offer a wide range of bee-essential nutritional components. The nutritional profile of pollen from different plant species varies, and selection by foraging adults is important when balancing the nutritional content of pollen fed to larvae. Improved understanding of their nutritional requirements is therefore important when selecting plant species for conservation habitats. Protein is an important component of bee diets and earlier work has linked amino acid levels in larval pollen diets with bee colony/population success. A threshold level has been proposed below which bumblebee colony development is poor, with significant improvement when exceeded, but little further advantage being gained by further increases thereafter. Dietary requirements differ between bee species/taxonomic groups, and this explorative study investigates the rationale behind dietary effects on solitary bees, focusing on the impact of the amino acid content of larval diets on the developmental success/survival of *Osmia bicornis,* the relevance of putative amino acid thresholds to the species, and whether larval pollen provisions selected/collected by foraging adults result in higher survival rates than artificial constructs. The findings are discussed in conjunction with other nutritional factors.

## 1. Introduction

Most flowering plants have evolved adaptations for attracting pollinating animals, and bees are considered primary pollen vectors that provide a key ecosystem service in both wild and agricultural landscapes [1]. Major attractants include nectar and pollen, with bees differing from most other pollinating groups, as many species utilise them for both adult nutrition and larval provisioning [2,3,4]. Although they may not be as energy-limited as many other animals, due to extensive access to nectar, a high-energy food source, the collection of pollen is energetically expensive, as individual floral visits usually yield only small quantities [5]. This can present a greater challenge to groups such as solitary bees when compared with colony-forming species, due to the lack of a large number of workers contributing to brood provisioning, thus offering potential advantages to selective foragers [6]. Indeed, it has been shown that, in some species of solitary bees, naturally foraged multifloral pollen balls are associated with higher larval survivorship than single-species pollen balls [7].

Much work on the nutritional quality of pollen has focused on proteins (and their constituent amino acids), but the importance of the level and ratio of other nutritional components, such as lipids (including phytosterols), carbohydrates, minerals, starch, vitamins, flavonoids and carotenoids, are also recognised [8,9,10,11].

Pollen nutritional quality affects a wide range of parameters contributing to the individual, colony or population success of solitary and social bees. These include physiological effects, immune system function, egg production, larval weight, larval survival, larval ejection, adult body size and longevity [9,12,13,14,15,16,17]. Recent research has linked *Osmia bicornis* fitness-related life history traits (mortality, cocoon development, and imago body mass) to chemical element availability in larval food, clarifying mechanisms underlying the nutritional ecology, behavioural ecology, and population functioning of bees within the context of nutrient cycling in the food web [13].

Increasing experimental data are available on the amino acid content of pollen, which has been identified as an important driver of colony or population success in many species, alongside a range of other essential nutritional characteristics [8,10,18,19,20]. The essential amino acids (EAAs) arginine, histidine, isoleucine, leucine, lysine, methionine, phenylalanine, threonine, tryptophan, and valine are reported to be important for honeybees with isoleucine, leucine and valine needed in the largest quantities, and histidine, methionine and tryptophan in lowest quantities [2]. As some pollen species do not offer the full requirements of these, and other dietary components for many bee species, mixed species pollen diets are thought to be more favourable than mono species diets, thus botanically diverse pollen mixes are recommended when pollinator-promoting habitats are established [14,17,21,22].

The characteristics of the relationship between larval survival/development and the amino acid content of larval diets are, however, not fully understood. Following microcolony studies of the bumblebee *Bombus terrestris audax*, Ryder et al. [9] postulated that, to avoid the detrimental effects associated with limited availability, favourable pollen mixes should exceed thresholds for nine essential amino acids, although no evidence of fitness differences above these thresholds was found. If they are confirmed to occur in various bee taxonomic groups, amino acid thresholds may contribute to a quantitative approach to the identification of useful plants to include in pollinator habitats, reducing current reliance on qualitative data [23], though they await confirmation.

Solitary bees of the genus *Osmia* are effective pollinators of important fruit crops worldwide, including *O. bicornis* in Europe, *Osmia cornifrons* in Asia, *Osmia cornuta* in Europe and North America, and *Osmia lignaria* in the USA [24]. *Osmia bicornis* (Hymenoptera: Megachilidae) is a univoltine polylectic species, which is distributed from Scandinavia to the Mediterranean and is active in Europe from February onwards, in synchrony with early season flowering periods [25,26,27]. Nests consist of a linear series of brood cells separated by mud walls, which are built in elongated cavities. A single egg is laid in each cell after it has been provisioned with sufficient pollen to sustain the development of all larval instars. The species is known to utilise flowers belonging to 19 plant families, providing individual larvae with the nutritional resources contained in mixtures of a variety of pollen species [7,28,29]. After hatching, larvae complete their development within the cell before spinning a four-layered cocoon in which adults develop, remaining in the cocoon throughout winter [25,30,31].

Improved understanding of the optimal chemical characteristics of pollens utilised by solitary bees relies on a wider knowledge of the chemical composition of bee-collected pollens, the physiology of developing bee larvae, the mechanisms of pollen digestion by pollen consumers, and the resultant impact on individual fitness [6,28]. This explorative study investigates the rationale behind the observed dietary effects on bees, focusing on whether the amino acid content of larval pollen provisions affects the developmental success and survival of the solitary bee *O. bicornis,* whether the concept that amino acid thresholds proposed for bumble bees may also be relevant to the species, and whether larval pollen provisions selected/collected by foraging adults result in higher survival rates than artificial constructs.

## 2. Materials and Methods

*Osmia bicornis* eggs were collected from artificial nest boxes (Mason Bees Ltd., Shrewsbury, Shropshire, UK) established on four lowland farms in three different agricultural regions of the UK (Shropshire: Halfway House/Great Wollaston, East Riding of Yorkshire; Dunswell, West Yorkshire; Rivelin Valley) that are representative of UK agricultural landscapes and climate. A survey of habitat types within 150 m flight radius around nest sites showed that they included floristically enriched field margins (both nectar-rich and wild bird seed plant mixes), grassland, winter wheat, winter barley, woodland and hedgerow. Full details and the surveying procedure establishing the presence and prevalence of herbaceous and woody plants are provided by Ryder [32]. Using a micro spatula, eggs were transferred from nest cells to a covered Petri dish containing damp filter paper and incubated under continuous darkness in a constant environment (CE) cabinet (LEEC, SL2, Nottingham, UK) at 5 °C/60% rH, for 7 days prior to the start of the experiment. Eggs from different sites were allocated to experimental treatments at random.

### 2.1. Treatments

Treatments (T1–T6) consisted of six different pollen mixes, including both single and multiple pollen diets, chosen to provide a range of amino acid content. One of the diets (T4) was collected by naturally foraging *O. bicornis* on a UK farm holding the LEAF Marque Standard [33]. This treatment is henceforth referred to as “wild-foraged”.

T1: Camellia (*Camellia* spp., Simianshan^®^, Jiangjin District, Chongqing, China).

T2: Phacelia (*Phacelia tanacetifolia*. Collected from a study plot not treated with pesticides (Biochem Agrar GmbH, Machern, Germany).

T3: A sweet chestnut (*Castanea sativa*)-dominated mix (TOCA^®^, Coruña, Spain), henceforth referred to as the “sweet chestnut mix”. Palynological analysis prior to use in the experiment found the sample to contain pollen from multiple plant species.

T4: Wild-foraged (collected from the *O. bicornis* larval cells from which eggs were sourced at the LEAF Marque site). The host farm participated in the UK Higher Level Environmental Stewardship Scheme [34] and maintained areas sown with seed mixes designed to provide plant communities rich in pollen and nectar for pollinators.

T5: Oilseed rape (OSR, *Brassica napus*, Simianshan^®^, Jiangjin District, Chongqing, China)

T6: Pine pollen (*Pinus spinosa*, Simianshan^®^, Jiangjin District, Chongqing, China).

All pollens were homogenised using a wet and dry grinder (Andrew James Ltd., Benfleet, Chelmsford, UK) and stored at −20 °C prior to use in the experiment. Due to the low water content of pollen, no adjustment was made for water content.

Pollens used in the artificial constructs were selected from commercially available species that are commonly found in farmland, woodland or in garden environments within close proximity of the selected sites in the UK. The pine pollen treatment was selected as a negative control, to investigate whether a pollen that is frequently not reported in *O. bicornis* larval diets, while still being available on the outside of the anther for foragers to collect, offers a low amino acid content which may be one factor contributing to its rejection as a forage source.

### 2.2. Palynological Analysis

Three samples were taken from each treatment to confirm the pollen composition. The percentage of each pollen species present was established under 400× magnification (TEC Microscopes Ltd., Stevenage, Welwyn Garden City UK), from a minimum sub-sample of 50 randomly selected grains [9,35]. Grains were recorded as unknown where identification was not possible.

### 2.3. Amino Acid Analysis

Three lypholised sub-samples (5–10 mg) of the homogenised pollen used in each treatment were subjected to acid hydrolysis and their amino acid content determined by ion-exchange chromatography, according to the European Pharmacopoeia methodology [36] (limit of quantification 5 nmol, ISO/IEC 17025—General requirements for the competence of testing and calibration laboratories, 2005). The results were expressed as total amino acid content (both incorporated amino acids and those free in solution, g/100 g), except for creatine and creatinine which cannot be assessed using the method. Further, tryptophan and cysteine/cystine are lost during acid hydrolysis. The levels of nine essential amino acids in each treatment (arginine, histidine, isoleucine, leucine, lysine, methionine, phenylalanine, threonine, and valine) [2] were analysed separately along with total amino acid (TAA) levels, total essential amino acids (EAAs) and total non-essential amino acids (NAAs).

### 2.4. Experimental Procedure

Individual brood chambers (IBCs) were constructed, consisting of a beech wood block (40 mm × 40 mm × 22 mm (length, width, depth)) with an 8 mm × 8 mm × 22 mm channel milled into the upper surface to form an artificial nest cell [7,28]. Each cell was provisioned with excess (>500 mg) of one of the pollen mixes (treatments) and a single *O. bicornis* egg was placed on top. The open side of the cell was covered with a glass cover slip (Fisher scientific Ltd., Leicester, UK) secured with a polypropylene-based transparent tape (Sellotape, Hertfordshire, UK), to allow observation of brood development. The IBCs were then incubated in a constant environment cabinet (LEEC, SL2, Nottingham, UK) at 20 °C and 60% rH, and in continuous darkness. There were 22 replicates for each pollen treatment except for phacelia pollen (due to limited availability), for which there were 18 replicates.

Larval development and survival in each IBC was assessed at three-day intervals, and the dates on which the following events occurred were noted: egg hatch, commencement of feeding without defecating (larval stage 1), commencement of feeding and defecating (larval stage 2), and completion of the cocoon (defined as when the cocoon becomes opaque, preventing observation of the larva within [37]). The cocoons were then carefully extracted and weighed, before being returned to the IBC for further observation.

Following the completion of the cocoons in late autumn (15 November) the incubation temperature was progressively reduced to 5 °C over a period of 4 weeks (to avoid cold shock), then maintained until the following spring (22 February). Thereafter, the temperature in the CE cabinet was progressively increased to 20 °C over a period of four weeks. Other environmental conditions remained unaltered throughout the autumn–spring period (60% rH, continuous darkness). Cocoons were monitored weekly to confirm successful adult emergence. The sex of all adults (irrespective of survival) was identified using the method of Falk [38]. Insects that did not emerge were dissected from the cocoon prior to identification.

### 2.5. Statistical Analysis

Statistical analysis was conducted using R Studio 0.99.903 [39], with the KMsurv (v0.1-5), Survival (v3.2-11) and the datasets load (v2.1.0) packages. All data were tested for normality via histograms and Shapiro–Wilks analysis, and log or sqrt transformations were applied where required.

Data describing TAA, total NAA and total EAA content (g/100 g) of the pollen used in different treatments were compared using ANOVA in each case. Levels of the individual essential amino acids in each treatment were also compared using ANOVA to investigate any potential relationship with pollen type (Treatment). Tukey post hoc tests were used to confirm where significant differences between treatments occurred.

To investigate differences in *O. bicornis* performance between treatments, dates of egg hatch, start of feeding, and start of defecation were expressed as the average of the two assessment dates between which the respective event occurred. Due to the nature of the ranked larval development data, a Scheirer–Ray–Hare nonparametric test was used to investigate the time to completion of the cocoon. Post-hoc Dunn tests for each significant factor or interaction were conducted.

Data comparing pupal weight at cocoon formation with bee sex and pollen treatment were subjected to ANOVA.

Larvae that had completed their cocoon were defined as survivors. Kaplan–Meier survival statistics were used to compare larval survival on the different pollen diets.

The number of days between egg hatch and completion of the cocoon was considered as ‘censored data’: individuals that died before the completion of the cocoon represented the exact observations for which the event (death) occurred, while those that completed the cocoon were the censored observations. A generalised linear model (GLM) with binomial error structure was constructed to test for differences between survival distributions. Factor reduction was conducted using the standard step-wise deletion procedure and non-significant terms and interactions removed to reach the minimum adequate model for all statistical tests. Following normal conventions, at each step of factor reduction, ANOVA between models was used to verify that the validity of the overall statistical model was not affected [40]. Any eggs that failed to hatch were excluded from the analysis.

## 3. Results

### 3.1. Palynological Analysis of Pollen

Palynological analysis confirmed that, in four treatments (the commercially sourced camellia (T1), oilseed rape (OSR) (T5), pine (T6) pollens, and the phacelia pollen obtained from the untreated field plot(T2)) only the expected pollen species were recorded (Table 1). The sweet chestnut pollen (T3) contained 66% *C. sativa*, with the remaining 34% consisting of pollen grains from three other plant species. Analysis of samples of wild-foraged UK pollen collected by *Osmia* (T4) showed that 85% of the larval resource consisted of *Ranunculus repens* or *Crataegus monogyna*, with three different plant species represented amongst the remaining pollen grains (Table 1).

### 3.2. Amino Acid Content of Larval Pollen Resources

**Figure 1:**
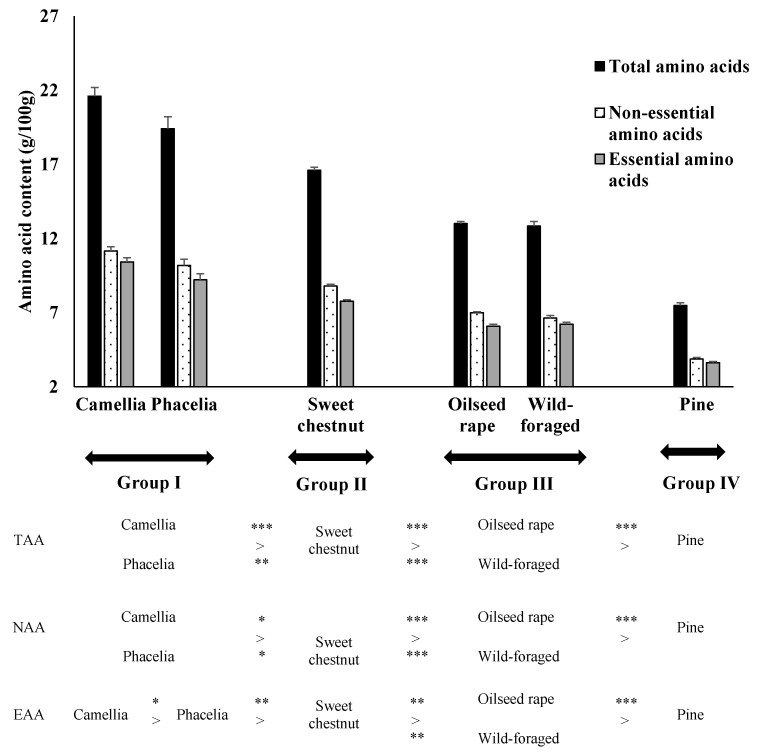
Amino acid content (mean ± S.E., g/100 g) of pollen resources offered to *O. bicornis* larvae in each of six treatments (camellia, phacelia, sweet chestnut, oilseed rape, wild-foraged and pine). Results for total amino acid content (TAA), total non-essential amino acid (NAA) content, and total essential amino acid (EAA) content can be divided into four statistically distinct groups. Pollen treatments in the same column are not statistically different (> direction of difference between columns, for differences between treatments * *p* < 0.05, ** *p* < 0.01, *** *p* < 0.001).

Amino acid content (either TAA, NAA or EAA) varied consistently between pollen treatments and could be divided into four groups (Figure 1). Less consistent differentiation between “Group II and III” treatments was identified when individual EAAs were investigated, suggesting a higher degree of overlap between the levels of some individual amino acids.

Total amino acid content was found to be normally distributed and there was a significant difference between treatments (F = 131.2, d.f. = 5.12, *p* < 0.001). Tukey post hoc tests confirmed that the TAA content of camellia and phacelia did not differ significantly, but both were higher than those found in all other treatments (Figure 1). Levels in the wild-foraged pollen and OSR treatments also did not differ significantly but were lower than in the sweet chestnut treatment, and higher than in the pine pollen.

Total NAA content was normalised by log transformation prior to ANOVA. There was a significant difference between treatments in terms of total NAA content (F = 230.1, d.f. = 5.12, *p* < 0.001). Levels of NAAs did not differ significantly between camellia and phacelia pollen, and both were higher than in all other treatments (Figure 1). Once again, levels in the wild-foraged pollen and OSR treatments did not differ significantly but were lower than in the sweet chestnut treatment, and higher than in the pine pollen.

Total EAA was normally distributed, and ANOVA identified a significant difference in levels between the pollen treatments (F = 119.91, d.f. = 5.12, *p* < 0.001). Tukey post hoc tests confirmed that camellia pollen had a higher level of EAAs than phacelia (Figure 1), which in turn contained higher levels than all other treatments. A significantly higher EAA content was found in the sweet chestnut pollen than in OSR, wild-foraged and pine pollen. Wild-foraged and OSR pollen did not differ in essential amino acid content, but both had higher levels than was recorded in pine pollen.

### 3.3. Individual Essential Amino Acids

Levels of individual essential amino acids (g/100 g) were normalised using square root transformation prior to ANOVA. There was a statistically significant interaction, namely, that the concentrations of individual EAA differed between pollen types; however, differences between EAA were not consistent between pollen types (F = 9.295, d.f. = 40.108, *p* < 0.001), thus differences between pollen types cannot be generalised for all EAA’s combined.

**Figure 2:**
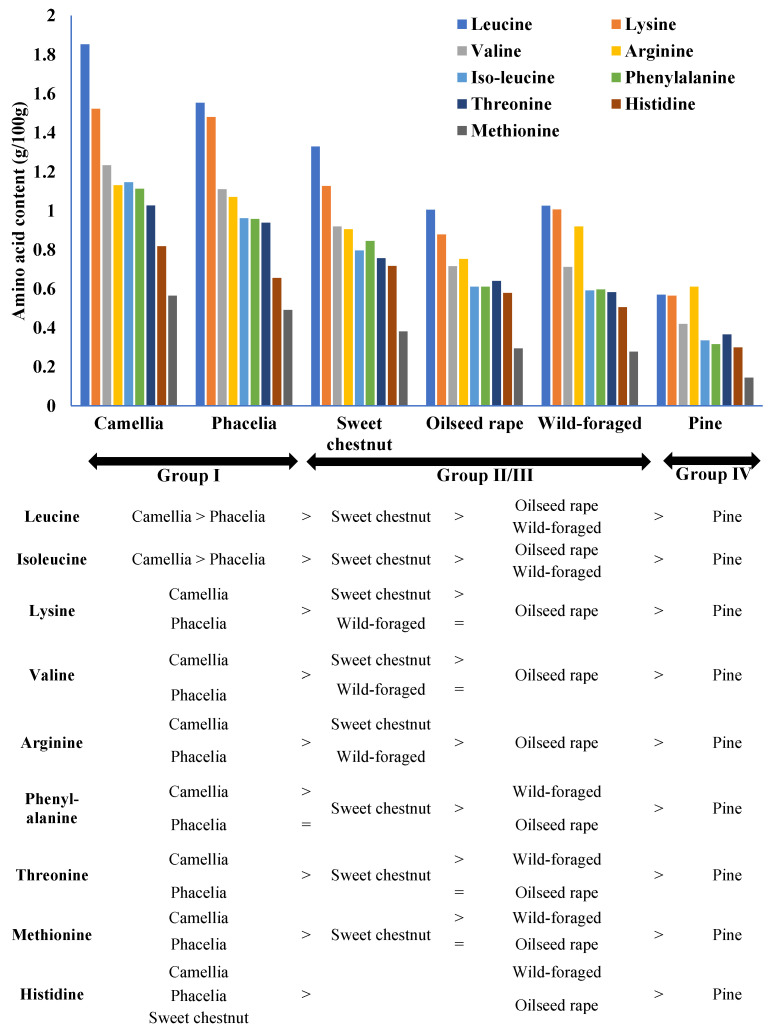
The levels of nine essential amino acids (mean ± S.E., g/100 g) in pollen resources offered to *O. bicornis* larvae in each of six treatments (camellia, phacelia, sweet chestnut, wild-foraged, oilseed rape, pine pollen) comprising the four groups defined in Figure 1. Pollen treatments in the same column are not significantly different, > indicates the direction of significant differences between columns, = indicate exceptions where no difference occurs between individual treatments between columns. (For full details of the levels of each statistical difference see Appendix A).

Individual essential amino acid content varied between pollen treatments and could be related to the four broad groups defined above (Figure 2). Comparison between treatments showed that significantly higher levels of each individual amino acid investigated (except phenylalanine in phacelia) occurred in the camellia and phacelia pollens (Group I) when compared with all other treatments, with the lowest levels in pine pollen (Group IV). Intermediate levels of each of the nine EAAs were recorded in Groups II (sweet chestnut) and III (OSR, wild-foraged pollens) treatments, however a less clear differentiation between these two groups was found. For example, significantly more (*p* < 0.001) leucine, isoleucine, phenylalanine, threonine, methionine and histidine were recorded in the sweet chestnut treatment (Group II) than in the wild-foraged treatment (Group III), whereas similar levels (*p* > 0.05) of lysine, valine and arginine were recorded. Statistically similar levels (*p* > 0.05) of all but one of the individual amino acids investigated were found in the wild-foraged and OSR treatments (both Group II), with higher levels of arginine (*p* < 0.05, see online resource Appendix A) in the wild-foraged pollen treatment.

### 3.4. O. bicornis Development and Survival

With pollen diets in which larvae survived until pupation, no effect of treatment on total larval development time between egg hatch and pupation (H = 11.06, d.f. = 5, *p* > 0.05) or any interaction between treatment and time (H = 9.69, d.f. = 29, *p* > 0.05) were recorded. Thus, no significant treatment differences were identified between individual components of larval development time (date of egg hatch, commencement of larval stages or date of cocoon completion). Pupal weight at cocoon formation was found to be independent of the bees’ subsequent sex, or pollen treatment (F = 0.081, d.f. = 4.31, *p* > 0.05). All pupae survived to eclosion in all treatments.

**Figure 3:**
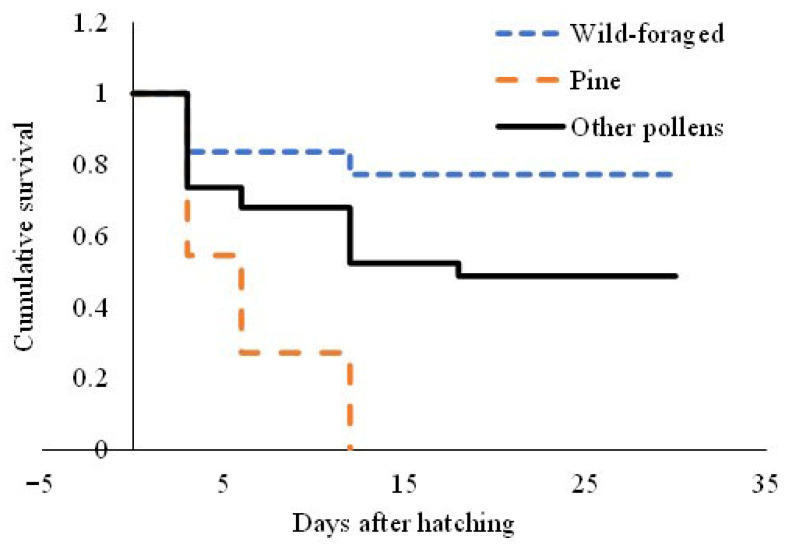
Cumulative survival of *Osmia bicornis* larvae when offered six different pollen diets. Mortality did not vary significantly between camellia, phacelia, OSR and sweet chestnut pollen treatments and these were combined into a single factor (“Other pollen mixes”) for analysis.

Larval survival of *O. bicornis* (defined as survival from egg hatch to successful pupation) differed significantly between the pollen diets (Kaplan–Meier analysis, log-rank test, x^2^ = 34.94, d.f. = 2, *p* < 0.001, Figure 3). During construction of the GLM investigating potential differences between survival distributions of immature stages fed using diets with different amino acid contents, no significant differences occurred between the survival of larvae offered camellia (T1), phacelia (T2), sweet chestnut pollen (T3) or OSR (T5). Thus, following normal practice [40], these were combined into a single factor for analysis (irrespective of whether the individual diets consisted of single or multiple pollen species), hereafter referred to as “other pollen mixes”. A significantly higher survival rate was recorded when wild-foraged UK pollen (T4) was offered (Z = −5.36, d.f. = 2, *p* < 0.001). No larvae survived in the pure pine pollen (T6) treatment (Z = −5.36, d.f. = 2, *p* < 0.001).

## 4. Discussion

Conservation habitats with increased flowering plant diversity are widely accepted as an effective approach to addressing issues of reduced biodiversity, and associated declining pollinator populations and ecosystem services [41,42]. The impact of such habitats for bee conservation depends, in part, on the provision of the multidimensional nutritional requirements of bee species, through the inclusion of a range of plant species that collectively provide the essential components of the required diets. Inconsistent advice on plant selection, however, often arises through many of the current comparisons of pollinator-attractive species being based on qualitative assessments of bee behaviour, with quantitative screening impeded by the potential cost of testing a sufficient proportion of candidate plant species [23]. An improved approach to plant selection would benefit from a wider understanding of the optimal balance of nutritional components required by different bee species, but in turn establishment of this balance relies, in part, on a knowledge of the precise requirements for individual components. Such understanding would establish a basis for ensuring that plant mixes in conservation habitats include sufficient sources of each component throughout the foraging seasons of the insects.

Larval pollen resources contain a range of essential nutritional components, including macronutrients (such as proteins and their constituent amino acids, carbohydrates, lipids (including phytosterols), vitamins, sterols, starch, and minerals), micronutrients (potassium, zinc, sodium), and secondary metabolites such as carotenoids and flavonoids [10,12,13,18,19]. Larval pollen diets also contain small amounts of nectar, primarily providing carbohydrates such as the disaccharide sucrose, and the monosaccharides glucose and fructose, together with other organic and inorganic compounds [42,43,44]. The relative importance of each of these components is related to species-specific nutritional requirements (including level and ratio), which can also vary between life stages (with adults often requiring more carbohydrates and larvae higher protein levels [21,45]). Many aculeate hymenopterans regulate their nutritional intake in an environment offering complex pollen choices through selective foraging by adults, although bee larvae may also contribute to regulating pollen consumption within the limitations imposed by the pollen ball provided by adults [12]. Whilst amino acid effects cannot be isolated from the general nutritional composition of the pollen diets, understanding of the combined effect of these multiple nutritional factors in the larval diet first requires an appreciation of the mechanisms underpinning the impact of individual components as a basis for future study. This study focuses on one of the components, amino acids, and their potential contribution to differences in larval survival. Interpretation of the importance of the amino acid content of the larval diet relies on consideration of the potential contribution of multiple, potentially interacting, nutritional drivers (such as, amongst others, carbohydrate availability, sterol composition, micronutrients, and pollen digestibility), particularly in the case of the wild-foraged pollen.

This explorative study investigates the rationale behind the effects of the amino acid content of individual pollen species and multi-species mixes, on survival of the solitary bee, *O. bicornis*. Amino acid and/or carbohydrate content of pollen are thought to be important drivers of fitness in a range of bee species [8,12]. Studies of survival and development have suggested that larval *O. bicornis* benefit from a comparatively high carbohydrate-biased diet and can tolerate a wide range of protein levels [12]. Further investigation of larval performance in relation to protein content of their food is required, however, to advance our understanding of how essential nutritional requirements are balanced in larval food and the broad tolerance for protein content.

The levels of ten specific amino acids are known to be critical for successful development of the larvae of honeybees (*Apis mellifera* [2,18]). A recent study of bumblebees has concluded that favourable pollen mixes for larval diets should provide a minimum level of at least nine EAAs to avoid egg laying, nest building, and thus colony biomass gain, being negatively affected [9]. However, no further advantage was recorded when levels exceeded these minimum putative “thresholds”.

The current work investigated the response of *O. bicornis* to the same range of individual EAAs, together with total protein, total non-EAA, and total EAA content. The levels of each varied consistently between the pollen resources offered to larvae, with the highest contents recorded in the camellia and phacelia treatments, the lowest in the pine pollen treatment, with the sweet chestnut-dominated pollen (containing four pollen species), OSR and the wild-foraged UK pollen (five pollen species) displaying intermediate levels. None of the *O. bicornis* larvae fed with pine pollen (containing the lowest amino acid content) survived to pupation, whereas significant survival was associated with all other larval diets. The outcome of this work therefore provides corroboration for the concept of putative thresholds existing for some bee species [9], below which a significant negative impact on larval fitness occurs but above which no additional advantage is accrued. However, the pollen diets are likely to have multidimensional variation in other components of the unmeasured nutritional axes, which will have contributed to the outcome. Similarly toxic substances within the pine pollen diet may have contributed to survival. The results therefore provide corroborative evidence of the existence of the proposed amino acid thresholds in *O. bicornis*, similar to those reported in bumblebees, and justify further controlled manipulation studies (similar to those of Filipiak and Filipiak and Austin and Gilbert [12,13]) to disentangle other influences before the concept can be confirmed.

Comparing diets that were above the putative amino acid/protein threshold, the wild-foraged pollen mix collected by adult *O. bicornis* was found to be associated with the highest rate of larval survival. However, the other diets tested, including the sweet chestnut mix (which displayed similar levels of plant species diversity to the wild-foraged UK pollen) and OSR pollen (both displaying similar amino acid content to the wild-foraged UK pollen mix), and the camellia and phacelia pollen (with higher levels of amino acids), each resulted in similar, lower survival rates. Thus, the higher survival recorded when larvae were offered wild-foraged pollen also supports the conclusion that other nutritional components in the multidimensional diet of the larvae may have contributed to the outcome. Austin and Gilbert [12] report that *O. bicornis* larvae self-select a carbohydrate-biased diet when compared with other hymenopterans and tolerate a broad range of protein levels, possibly the result of a low level of nectar in pollen balls supplied by adults and the relatively high protein levels in the pollen. The results of our study suggest that the lack of improved fitness when protein levels in the diet exceed a putative minimum level may facilitate the reported tolerance. The increased fitness when fed the naturally foraged diet suggests that nectar may be an important component, and that adults may actively select more favourable pollens from which the larvae self-select to balance nutritional intake, supporting the conclusions of the earlier studies [9,12]. Further research should focus on more precisely controlled manipulations of larval diets [12,13] to more accurately identify the putative thresholds identified in this explorative experiment.

## Figures and Tables

**Table 1:** The mean proportion of pollen grains from different plant species present in larval resources used in the treatments for the *O. bicornis* performance experiments.

Treatment Name	Genus/Species Content	Mean Proportion
Camellia Pollen (T1)	*Camellia* spp.	1.00
Phacelia (T2)	*Phacelia* spp.	1.00
Sweet chestnut (T3)	*Castanea sativa*	0.66
	*Prunus* spp.	0.17
	*Vicia* spp.	0.10
	*Brassica napus*	0.07
Wild-foraged (T4)	*Ranunculus repens*	0.60
	*Crataegus monogyna*	0.25
	*Centaurea nigra*	0.10
	*Vicia* spp.	0.01
	*Rubus fruticosus*	0.04
Oilseed rape (OSR, T5)	*Brassica napus*	1.00
Pine (T6)	*Pinus spinosa*	1.00

## Data Availability

The original contributions presented in the study are included in the article, further inquiries can be directed to the corresponding author(s).

## Appendix A

Outline Method (full details are provided in the associated paper): Pollen treatments included: chestnut, camellia, oilseed rape (OSR), pine pollen, phacelia, and pollen foraged by *O. bicornis* and used to resource their larval cells (see paper for full details).

The amino acid (AA) content of pollen from three (5–10 mg) sub-samples of each pollen treatment was determined after acid hydrolysis of lypholised samples using the European Pharmacopoeia methodology. The levels of nine essential AAs in each treatment (arginine, histidine, isoleucine, leucine, lysine, methionine, phenylalanine, threonine, valine; g/100 g), were investigated individually. Following square root transformation, data were subjected to ANOVA and Tukey post hoc tests to confirm where significant differences between treatments occurred.

Arginine: Camellia and phacelia pollen contained similar levels of arginine, which were higher than in all other treatments (*p* < 0.05). Arginine content of *O. bicornis*-foraged and chestnut pollen did not differ, both having higher levels than was recorded for OSR pollen (*p* < 0.05). Pine pollen had a lower arginine content than chestnut, *O. bicornis*-foraged (*p* < 0.001) and OSR (*p* < 0.05).

Histidine: A similar histidine content was recorded in the camellia, phacelia and chestnut pollen samples, and higher histidine levels were found in the *camellia* and *phacelia pollen* compared with all the remaining treatments (*p* < 0.01). The chestnut pollen contained a higher level of histidine than was recorded in the OSR (*p* < 0.05), *O. bicornis*-foraged and pine pollens (*p* < 0.001). The histidine content of the *O. bicornis*-foraged and OSR pollens did not differ from each other, but both had higher levels of histidine than in the pine pollen (*p* < 0.001).

Isoleucine and leucine: Camellia pollen had higher levels of leucine and isoleucine compared with all other treatments (*p* < 0.01). Phacelia contained higher levels than all other treatments except camellia (*p* < 0.01). More leucine and isoleucine were recorded in chestnut pollen than in OSR, foraged pollen and pine pollen (*p* < 0.001). The amount of the two AAs recorded did not differ between *O. bicornis*-foraged and OSR pollen, but both contained higher levels than were found in pine pollen (*p* < 0.001).

Lysine and valine: Camellia and phacelia pollen had similar lysine and valine content, but higher levels of both AAs compared with all other treatments (*p* < 0.001). Pine had lower levels than chestnut, foraged pollen and OSR (*p* < 0.001). Lysine and valine content of *O. bicornis*-foraged pollen did not differ from that of chestnut or OSR pollen, but chestnut had higher levels than OSR (*p* < 0.001).

Methionine and threonine: Methionine and threonine levels in camellia and phacelia pollen did not differ, and higher levels were present in both when compared with all other treatments (*p* < 0.05). Chestnut pollen had higher levels than were recorded in the *O. bicornis*-foraged and pine pollens (*p* < 0.001), but was similar to OSR. *Osmia bicornis*-foraged and OSR pollens did not differ in threonine and methionine content, but had higher levels than pine pollen (*p* < 0.001).

Phenylalanine: Camellia and phacelia pollen did not differ from each other in phenylalanine content. Camellia had significantly higher phenylalanine levels than all other treatments (*p* < 0.05). Levels in phacelia and chestnut were similar, but chestnut pollen contained more phenylalanine than OSR, *O. bicornis*-foraged and pine pollen (*p* < 0.001). *Osmia bicornis*-foraged and OSR pollen did not vary in phenylalanine content compared with each other, but both had higher levels than was recorded in pine pollen (*p* < 0.001).
